# Intimate partner violence and utilization of maternal health care services in Addis Ababa, Ethiopia

**DOI:** 10.1186/s12913-017-2121-7

**Published:** 2017-03-07

**Authors:** Bedru Hussen Mohammed, Janice Mary Johnston, Joseph I. Harwell, Huso Yi, Katrina Wai-kay Tsang, Jemal Ali Haidar

**Affiliations:** 1 School of Public Health, The University of Hong Kong, G09, G/F, Patrick Manson Building, 7 Sassoon Road, Pokfulam, Hong Kong; 2 Clinton Health Access Initiative, Boston, USA; 3 JC School of Public Health and Primary Care, Chinese University of Hong Kong, Hong Kong, China; 4 School of Public Health, Addis Ababa University, Addis Ababa, Ethiopia

**Keywords:** Intimate partner violence, Maternal health care, Antenatal care, Prevention of mother-to-child transmission, Addis Ababa

## Abstract

**Background:**

Despite its prominence, intimate partner violence (IPV) against women has received little attention in Ethiopia. And as many of sub-Saharan African countries, maternal health care services utilization remains poor. Full access and utilization of maternal health care services is a key to significant reduction in maternal and child mortality, and eliminate new HIV infection in infants. Identifying the factors that contribute to the poor access and utilization should aid the design of appropriate policy and intervention strategies. Thus the objective of this study was to examine the association between IPV and use of maternal health care services in Addis Ababa, Ethiopia.

**Methods:**

A cross sectional study on couples (N = 210; male/female pairs) with an infant less than 6 months of age was conducted. The dependent variable was use of maternal health care services and the main independent variable was IPV. Data was collected using face-to-face self-reported questionnaires and analyzed using SPSS version 20.0. Bivariate and multivariate logistic regression models were used to examine the relationship between the dependent and independent variables.

**Results:**

The mean age of the women was 28.7 years (SD = 5.4), on average women were 7.4 years (SD = 7.4) younger than their partners. Although most of the women (95.2%) had at least one antenatal care (ANC), only 35 (2%) had ≥4 ANC visits and about half (49.0%) had their first ANC visit within the first trimester. Women who experienced emotional IPV in their relationship were less likely to have their 1^st^ ANC within three months of pregnancy (AOR = 0.69; 95%CI = 0.49–0.96). Women who reported physical IPV in their relationship were less likely to use ≥4 ANC (AOR = 0.48; 95%CI = 0.21–0.71), be tested for HIV (AOR = 0.26; 95%CI = 0.09–0.79), have skilled delivery attendant (AOR = 0.31; 95%CI = 0.12–0.98), and deliver in a health facility (AOR = 0.35; 95%CI = 0.14–0.88). Likewise, women experienced sexual IPV or partner control in their relationship were less likely to use ANC ≥4 times (AOR_sexual-IPV_ = 0.91; 95%CI = 0.84–0.98 and AOR_partner-control_ = 0.38; 95%CI = 0.17–0.85 respectively).

**Conclusions:**

IPV is prevalent among couples in Addis Ababa, Ethiopia where three out of four women reported having experienced one or more type of IPV in their current relationship. And all types of IPV showed significant association with poor utilization of one or more maternal health care services. Thus efforts to sustain the recent success in maternal health and further improvement should give due consideration to IPV.

**Electronic supplementary material:**

The online version of this article (doi:10.1186/s12913-017-2121-7) contains supplementary material, which is available to authorized users.

## Background

The Millennium Development Goal (MDG) five was targeted to improve maternal health by reducing maternal mortality and universal access to reproductive health by 2015 [1]. Although substantial progress has been made globally, outcomes in a number of developing countries is limited [2]. More recently adopted the Sustainable Development Goals (SDG) targets reducing the global maternal, neonatal and under-five mortality, and ending the epidemics of HIV/AIDS by 2030 [3], and achieve gender equality and empower all women and girls [4].

Ethiopia has recorded some progress in maternal and child health in the past decades: under-five mortality rate declined from 198 deaths per 1,000 live births in 1990 to 88 in 2011 [5]. Despite this improvement, maternal health care access and utilization in general is still poor, even compared to some sub-Saharan African countries [6]. Only 34% of women had at least one ANC visit, and women who had four or more visits were only 19%, and only 12% of deliveries attended by skilled by health professionals [7].

Violence against women is a major public health problem, and intimate partner violence (IPV) is the most common form of violence against women [8–10]. IPV against women is a universal phenomenon that exists in all countries of the world [11]. Globally, almost one third (30%) of women who have been in a relationship report that they have experienced some form of physical and/or sexual violence by their intimate partner [11]. In sub-Saharan Africa the prevalence is estimated to be between 20% and 70% [11]. A 2005 study by WHO, reported that Ethiopia has the highest rates of IPV compared to the ten countries in the study [12]. 53.7% Ethiopian women had experienced IPV (either physical or sexual or both) within one year preceding the interview and 70.9% over their lifetime [12]. The same study also reported that 49% and 59% of women experienced physical and sexual violence by a partner at some point in their lives, respectively.

Ethiopia is a culturally diverse with institutionalized gender roles and structural power imbalances between women and men [13]. This makes IPV not only a deep-rooted problem but also somehow accepted rather than challenged. 45% of men and 68% of women took part in the 2011 Ethiopian Demographic and Health Survey (EDHS) reported that wife beating is justified [6].

IPV has been linked to adverse health outcomes [14–17]. Studies have also shown that women’s experience of physical violence is significantly associated with low use of contraception, sexually transmitted diseases (STDs), unwanted pregnancy, antenatal hospitalization, repeat pregnancy, miscarriages, low birth weight, preterm delivery, and neonatal and child mortality [18–22]. Experience of physical IPV is also reported to be associated with lower use of sufficient ANC and assisted deliveries from a skilled provider [23]. Two studies in Kenya also showed that IPV is linked with not having skilled attendance during delivery [24, 25].

Although studies have identified some forms of IPV are associated with the use of some maternal health care services, the role that some forms of IPV play in the utilization of maternal health care services has received less attention. Most studies focused on the relationship between physical and sexual IPV and health outcomes [23]. Little is known about the link between emotional IPV and partner controlling behavior with utilization of maternal health care services.

Better understanding of the association between all the four forms of IPV and use of maternal health care services is important especially in a highly patriarchal society like Ethiopia, where culture and societal norms allow men to make decisions on the issue of women’s health [6]. It provides a policy tool for designing interventions to address maternal health care needs in the country. Thus, the aim of this study was to examine the association between IPV and maternal health care services utilization in Addis Ababa. The study hypothesized that all forms of IPV are associated with lower likelihood of maternal health care services utilization.

## Methods

### Study design and setting

The data for this study was drawn from a larger couple community based study examining access and utilization of maternal and child health in Addis Ababa, Ethiopia. Two factors determined the selection of Addis Ababa as the study site. It has a diverse population representing almost all ethnicity and culture, and it has higher HIV prevalence (6.0%) than the national urban average (5.2%) [6].

### Sample size and sampling technique

Sample size was calculated using a single population proportion sample size estimation formula [26]: *N* = [(*Z*_α/2_)^2^ *p* (1- *p*)]/*d* ^2^. Since there is no single indicator for maternal and child health care services, the sample size calculation assumed a 93.6% proportion (p) considering receiving ANC from a skilled provider (doctor, nurse or midwife) as use of maternal and child health care service [6]. A standard normal variate [*Z*_α/2_^2^ at 5% type 1 error (*p* < 0.05)] of 1.96; 5% absolute precision (*d*); 95% confidence level; 1.5 design effect; and 72% expected response rate were taken to calculate the sample size which resulted in a sample size of 210 couples.

Multi-stage cluster sampling procedure was used to select representative sample. The city has ten sub-cities divided into 116 districts (Woredas). All sub-cities were included in the study. Then districts of each sub-city were listed and one district was selected randomly from each sub-city. Then, couples fulfilling the inclusion criteria were randomly selected from the list of couples in the ten selected districts until the required sample size were fulfilled. All eligible couples in the selected districts had the same probability of being selected.

The study included couples living in a relationship (married or cohabiting), had a child in the last six months, lived together during the period of the pregnancy and resided in Addis Ababa at least for the past one year.

### Study variables

The study used two, male and female, types of questionnaires (Additional files 1 and 2). The outcome variable used for this particular study was maternal health care services utilization. Since maternal health care has cascades of services the study used items to assess it. The items included are ever attend antenatal clinic during the pregnancy; time of first ANC visit; number of ANC visits throughout the pregnancy; testing for HIV during the pregnancy; delivery with skilled birth attendant; and delivery in a health facility.

IPV was the main explanatory variable and is measured as experience of violence by women perpetrated by their current male partner. The study adopted the tool used in the WHO multi-country study on women’s health and domestic violence against women to measure the four forms of IPV: emotional, physical, sexual, and partner control [12]. The tools have been validated in a variety of cultural settings including Ethiopia [27], and has a series of direct and clearly worded ‘yes’ or ‘no’ questions.

To assess emotional violence, women responded to four questions including: did your partner ever insulted or made feel bad about herself; belittled or humiliated in front of other people; done things to scare or intimidate her on purpose; or threatened to hurt her or someone she cares about. Similarly, women responded to six questions measuring physical violence including: did your partner ever slapped or thrown something at that could hurt; pushed or shoved or pulled hair; hit with a fist or with something else that could hurt; kicked, dragged, or beaten up; choked or burnt on purpose; or threatened to use or actually used a gun, knife, or other weapon against you. And three questions related to sexual violence including: did your partner ever physically forced you to have sexual intercourse against your will; have you ever had sexual intercourse because you were afraid of what your partner might do to you; and have you ever been forced by your partner to do something sexual you found degrading or humiliating.

Partner controlling behavior was assessed using six questions about whether the male partner had control issues. Women were asked if their current partners: get jealous if she talks with other men, accuses her of unfaithfulness, does not permit her to meet her friends, tries to limit her contact with family, insists on knowing where she is at all times, and doesn’t trust her with money.

There were also questions about the timing of the violence, allowing analysis of the extent to which different forms of violence occurred in the 12 months prior (during pregnancy) versus during the whole relationship with the current partner.

The study also assessed women’s attitude towards male partners’ physical violence. It was measured by a five item scale which is commonly used in household surveys, including Multiple Indicator Cluster Surveys (MICS) and Demographic and Health Surveys (DHS), in which women are asked whether they think a man is justified in hitting or beating his female partner under certain circumstances: if she goes out without telling him, neglects the children, argues with him, refuses to have sex with him, or she burns the food [12].

Other covariates that have been theoretically and empirically proven to be significantly associated with IPV and maternal health care were included in the analyses. These include; maternal age, parity, educational status of the woman and the partner, employment status, woman’s decision-making autonomy, weekly mass media exposure of women and household monthly income (the sum of monthly income of the couples). Women’s decision-making autonomy was measured by their involvement in making decisions, alone or with partner, regarding: (a) their personal health care, (b) large household purchases, (c) daily household purchases, and (d) family or relatives visits.

### Data collection

Data were collected for a period of five months, June 2014 to October 2014, in all selected study sites. The study used a team approach to data collection. The research team was composed of fifteen trained health extension workers (HEWs) actively working in the community of the selected districts at the time of data collection. A daylong training was given to the HEWs on the data collection technique before data collection started. They were aware of the community, which facilitated the selection of participants. The familiarity of the data collectors to the participants also gave them ability to establish good connection during the interview and put the respondents at ease. The primary investigator supervised the data collection. The filled questionnaires were checked for completeness by principal investigator upon collection from the data collectors.

### Statistical analyses

Data were entered and analyzed using the IBM SPSS Statistics 20 version. Descriptive statistics were used to describe the socio-demographic characteristics of the participants, IPV, and the utilization of maternal health care services.

The six items of maternal health care services utilization were binary variables and responses were grouped into two categories and coded 1; if a woman attended ANC, had first ANC visit within first trimester, had four or more ANC visits throughout the pregnancy, tested for HIV, assisted by a health professional during delivery, and delivered in a health facility; and 0 if not. Similarly, for IPV, women answered ‘yes’ to any one of the items under emotional, physical, sexual violence or partner control subscales were considered emotionally, physically, sexually abused or controlled, respectively. In addition the severity of a physically violent act was ranked according to its likelihood of causing physical injuries [12].

For bivariate analysis, frequencies and cross tabulations were used to identify the distributions of the outcome variables by selected background characteristics. The chi-square test of association was used to test the statistical significance of the differences in the use of maternal health care services. The level of significance was set at *p* < 0.05. The outcome measures are dichotomous variables and as such binary logistic regression models were used to examine the relationship between IPV occurrence during the relationship and utilization of maternal health care services.

A total of six models were estimated (one model for each outcome variable). This enabled the assessment of the association between each of the IPV types occurred in the current relationship and the maternal health care indicators while controlling for other covariates. The adequacy of each model was tested by the Hosmer and Lemeshow test for goodness of fit. The strength of the association for each logistic regression model was estimated using the adjusted odds ratio (AOR) and 95% confidence intervals (CI).

## Results

The number of women participated in the study was 210 and displayed in Table 1. The age of participants ranged from 18 to 42 years, with mean 28.7 (SD = 5.4). In 89.5% of couples the male partner was older than the female partner by one or more years. On average men were older than women by 7.4 years (SD = 7.4). The majority (60%) of participants were Orthodox, while more than one in four were Muslims. The proportion of women who had no formal education was 15.2%, which is higher than their male counter parts (9.5%). Nearly one-third of the women (32%) had job at the time of the interview.

**Table 1:** Descriptive statistics for socio-demographic characteristics of women and distribution according to different maternal health care services use in Addis Ababa, 2014 (N = 210)

Background Characteristics	All women n (%)	At least one ANC %	1st ANC within 1^st^ trimester %	Four or more ANC %	Tested for HIV %	Skilled delivery assistant %	Delivery in health facility %
Age in years							
18–25	50 (23.8)	98.0	58.0	48.0*	94.0*	92.0	86.0*
26–35	131 (62.4)	94.7	46.6	33.6	84.7	84.7	78.6
36–45	29 (13.8)	93.1	44.8	20.7	69.0	79.3	58.6
Age gap of couples							
partner younger or same age as women	21 (10.0)	100.0	61.9	33.3	90.5*	100.0	90.5*
partner older by 1–5 years	73 (34.8)	95.9	50.7	39.7	89.0	84.9	80.8
partner older by 6–10 years	64 (30.5)	96.9	51.6	42.2	90.6	87.5	82.8
partner older by more than 10 years	52 (24.8)	90.4	38.5	21.2	69.2	78.8	61.5
Ethnicity							
Amhara	78 (37.1)	96.2	56.4	44.9	87.2	89.7	80.8
Oromo	46 (21.9)	91.3	56.5	23.9	82.6	76.1	67.4
Tigre	22 (12.9)	96.3	40.7	25.9	88.9	81.5	81.5
Other	59 (28.1)	96.6	37.3	35.6	81.4	89.8	79.7
Religion							
Orthodox	128 (61.0)	96.9	56.2	39.1	85.9	85.2	77.3
Muslim	56 (26.1)	92.9	35.7	26.8	83.9	89.3	76.8
Other	26 (12.4)	92.3	42.3	34.6	80.8	80.8	80.8
Educational status							
no formal education	32 (15.2)	84.4*	12.5*	11.0*	62.5*	62.5*	46.9*
primary school	80 (38.1)	96.2	51.2	35.0	85.0	88.8	78.8
secondaryand above	98 (46.7)	98.0	59.2	46.9	91.8	90.8	86.7
Partner’s educational status							
no formal education	20 (9.5)	85.0	15.0*	15.0	60.0*	70.0*	40.0*
primary school	50 (23.8)	94.0	42.0	30.0	80.0	81.0	74.0
secondary and above	140 (66.7)	97.1	56.4	40.0	90.0	89.5	84.3
Employment status							
Employed	67 (31.9)	98.5	55.2	44.8*	92.5*	89.6	83.6
Unemployed	143 (68.1)	93.7	46.2	30.8	81.1	83.9	74.8
Household monthly income (ETB)							
Low income (</=1000ETB)	73 (34.8)	94.5	43.8	30.1	82.2	82.2	72.6
Middle income (1001–2000ETB)	70 (33.3)	95.7	50.0	34.3	85.7	91.4	81.4
High income (>2000ETB)	67 (31.9)	95.5	53.7	41.8	86.6	83.6	79.1
Couples relationship duration							
Less or equal to 4 years	91 (43.3)	97.8	48.4	34.1	96.7*	86.8	79.1
More than 4 years	119 (56.7)	93.3	49.6	36.1	75.6	84.9	76.5
Parity							
1	66 (31.4)	95.5	51.5	30.3	90.9	84.8	74.2
2–3	116 (55.2)	94.8	47.4	40.5	82.8	83.6	80.2
4+	28 (13.3)	96.4	50.0	25.0	78.6	96.4	75.0
Weekly mass media exposure							
No exposure to all	31 (14.8)	87.1*	29.0*	3.2*	64.5*	74.2	54.8*
Exposed to one type	77 (36.7)	93.5	46.8	27.3	84.4	83.1	77.9
Exposed to two types	78 (37.1)	98.7	53.8	43.6	91.0	89.7	82.1
Exposed to all three types	24 (11.4)	100.0	66.7	75.0	91.7	95.8	91.7
Women’s decision making autonomy							
No involvement in all	12 (5.7)	83.3*	25.0*	16.7	66.7	66.7	50.0
Involved in one	26 (12.4)	84.6	3.8	26.9	73.1	84.6	76.9
Involved in two	31 (14.8)	96.8	22.6	19.4	87.1	74.2	67.7
Involved in three	43 (20.5)	97.7	58.1	44.2	83.7	90.7	88.4
Involved in all four	98 (46.7)	98.0	68.4	40.8	89.8	89.8	79.6
Emotional IPV							
No	161 (76.7)	96.9	51.6*	39.8*	88.2*	87.0	80.7*
Yes	49 (23.3)	89.8	40.8	20.4	73.5	81.6	67.3
Physical IPV							
No	171 (81.4)	96.5	53.2*	42.1*	89.5*	88.9*	82.5*
Yes	39 (18.6)	89.7	30.8	25.1	64.1	71.8	56.4
Sexual IPV							
No	131 (62.4)	96.2	55.0*	44.3*	87.0	88.5	80.9
Yes	79 (37.6)	93.7	39.2	20.3	81.0	81.0	72.2
Partner control							
No	65 (31.0)	98.5	60.0*	52.3*	93.8*	93.8*	86.2*
Yes	145 (69.0)	93.8	44.1	27.6	80.7	82.1	73.8

*IPV* intimate partner violence occurred during the current relationship, *ANC*, antenatal care; Skilled health providers include doctors, nurses and midwives, Weekly mass media exposure = exposure to TV, Newspaper and Radio at least once a week; Women’s decision making autonomy = women’s participation alone or jointly in decisions regarding their personal health care, large household purchases, daily household purchases and family or relatives visit; ∗ = *p* < .05 significance level

Majority of the couples (96.7%) were married, and the mean relationship duration of the couples was 6.9 years (SD = 50). The mean number of children in the relationship was 2.2 (SD = 1.26, min = 1 and max = 9). The mean household monthly income of couples was about 2,215.00 Ethiopian Birr (ETB), which is equivalent to 103.00 USD, and more than one-third (35.4%) of households had monthly income of 1,000.00 ETB (46.00 USD) or less.

Of the total sample, 50.4% of women listened to the radio, and 22.4% reported that they watched television at least once in a week. However, about 85% women had no access to newspaper at least once in a week. Regarding women’s decision-making autonomy, 16.7% women reported that their male partners made decisions regarding their personal health care. Women had least autonomy in decisions concerning large household purchases (7.1%) than any of the activities. And they reported highest autonomy in making final decisions in daily household purchases (46.2%). Only 46.7% of women were involved in all four decisions and 5.7% had no involvement in all four decisions.

**Fig. 1:**
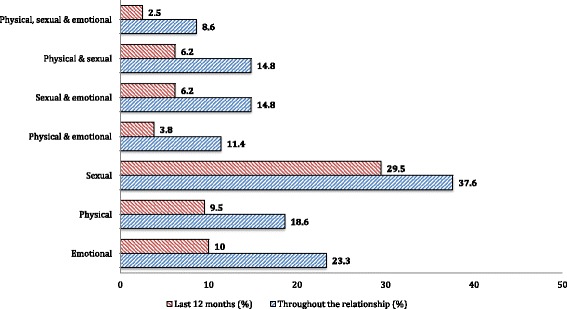
Prevalence of physical, sexual and emotional violence against women among couples in Addis Ababa, 2014 (N = 210)

Figure 1 shows women’s experiences of different forms of IPV. The most common act of IPV reported in current relationship was partner controlling, reported by 69.0% of women. The most common controlling behavior of male partner reported by participants was insisting to know the women’s whereabouts at all times, reported by 54.3% of women. Whereas, the least reported controlling behavior was not trusting with money (10.5%).

The second most common form of IPV was sexual violence, which was reported by 37.6% of the women; particularly having sexual intercourse with partner because of fear was reported by 34.3% of women. Emotional violence was the third common form of IPV reported by the women. At least one type of emotional IPV has happened in 23.3% of the women; being ever insulted or made to feel bad was the most common form of emotional IPV.

About one in five (18.6%) of the women had experienced at least one type of physical violence during their current relationship. The prevalence of severe physical violence among women was 9.0%. The proportion of women who has reported emotional, sexual, and physical IPV in the last twelve months were 10%, 29.5%, and 9.5%, respectively.

With regard to women’s attitude towards partners’ physical IPV, considerable proportion of the women believed that men are justified in hitting or beating their female partner if she refuses to have sex (35.2%) and if she goes out without telling him in advance (29.5%). But only 6.7% of women said men could hit or beat their women if she burns the food.

Utilization of maternal healthcare services was analyzed with respect to the most recent live birth the women had. Considerable proportion, 4.8%, of women had no ANC at all, and only 35.2% had four or more ANC visits. About half (49.0%) of women had their first ANC visit within the first trimester of their pregnancy. The mean gestational age of first ANC visit was 3.5 months (SD = 1.29). More than three fourth (77.6%) of women had delivery in a health facility and 85.7% of women delivered with the assistance of a skilled health care provider. And 84.8% of the women had tested for HIV during pregnancy.

The bivariate analyses revealed several significant differences in the use of maternal health care services across various socio-demographic groups (Table 1). These include women’s age, women’s educational status, partner’s educational status, couples’ age difference, women’s employment status, relationship duration, women’s weekly mass media exposure and women’s decision-making autonomy.

Table 2 presents the result of the adjusted logistic regression models performed to identify the IPV correlates of maternal health care services after controlling for other covariates (women’s age, couple’s age gap, women’s educational status, partner’s educational status, women’s decision-making autonomy, women’s employment status, women’s weekly mass media exposure, couple’s relationship duration and household monthly income). Women who experienced emotional IPV in their relationship were less likely to have their 1^st^ ANC within three months of gestational age (AOR = 0.69; 95%CI = 0.49–0.96). Women who have been physically abused by their partner were less likely to have: four or more ANC visits (AOR = 0.48; 95%CI = 0.21–0.71); HIV testing (AOR = 0.26; 95%CI = 0.09–0.79); skilled delivery attendant (AOR = 0.31; 95%CI = 0.12–0.98); and deliver in a health facility (AOR = 0.35; 95%CI = 0.14–0.88). In addition, the odds of using ANC four or more times was less in women who reported sexual abuse in their relationship as compared to those who haven’t (AOR = 0.91; 95%CI = 0.84–0.98). Lastly, the study result showed that women who experienced partner control in their relationship were less likely to use ANC four or more times during their last pregnancy (AOR = 0.38; 95%CI = 0.17–0.85).

**Table 2:** Adjusted logistic regression models (AOR) for the association between different forms of IPV in couples’ relationship and utilization of maternal health care services, Addis Ababa, Ethiopia, 2014 (*N* = 210)

IPV type	At least one ANC	1st ANC within 1^st^ trimester	Four or more ANC	Tested for HIV	Skilled delivery assistant	Delivery in health facility %
	AOR^#^ (95% CI)	AOR (95% CI)	AOR (95% CI)	AOR (95% CI)	AOR (95% CI)	AOR (95% CI)
Emotional						
No	1.00	1.00*	1.00	1.00	1.00	1.00
Yes	0.56 (0.10–3.13)	0.69 (0.49–0.96)	0.61 (0.24–1.52)	0.50 (0.17–1.48)	1.18 (0.41–3.39)	0.89 (0.36–2.18)
Physical						
No	1.00	1.00	1.00**	1.00*	1.00*	1.00*
Yes	0.78 (0.13–4.43)	0.68 (0.27–1.69)	0.48 (0.21–0.71)	0.26 (0.09–0.79)	0.31 (0.12–0.98)	0.35 (0.14–0.88)
Sexual						
No	1.00	1.00	1.00*	1.00	1.00	1.00
Yes	1.03 (0.88–1.20)	1.01 (0.95–1.08)	0.91 (0.84–0.98)	0.99 (0.90–1.08)	1.04 (0.95–1.13)	0.95 (0.88–1.03)
Partner control						
No	1.00	1.00	1.00*	1.00	1.00	1.00
Yes	0.78 (0.07–8.62)	0.97 (0.45–2.08)	0.38 (0.17–0.85)	0.45 (0.12–1.60)	0.45 (0.13–1.51)	0.70 (0.28–1.74)

*IPV* intimate partner violence, *ANC* antenatal care, *#* models adjusted for women’s age, couple’s age gap, women’s educational status, partner’s educational status, women’s decision-making autonomy, women’s employment status, women’s weekly mass media exposure, couple’s relationship duration and household monthly income; ∗ = *p* < .05; ∗∗ = *p* < .01; ∗∗∗ = *p* < .001

## Discussion

This study is one of the first studies reporting the association between IPV and maternal health care services utilization among pregnant women in Addis Ababa, Ethiopia.

The overall IPV prevalence rate during the current relationship was 75.2%. This finding was comparable with the 2005 lifetime prevalence of IPV prevalence report of the WHO multi-country study among women aged 15–49 years where 71% of women experienced physical and/or sexual IPV [12]. The most common form of IPV reported in this study was partner control (69.0%); followed by sexual violence (37.6%), emotional violence (23.3%) and physical violence (18.6%), respectively. When compared with the 2005 WHO report sexual, physical and emotional IPV prevalence in the study was lower and the difference could be partly explained by the fact that the previous report was lifetime IPV while this study examined IPV in the current relationship.

Women’s attitude towards partners’ physical violence was also alarmingly high, 52.4%. More than half of the women in the study believed that men are warranted to beat their women when if she refused to have sex with him, suggesting that IPV is tolerated by the women themselves lending to the higher IPV occurrence in this study. The finding was similar with study report by Abebe et al. [28]. Nevertheless, when compared with the earlier WHO multi-country study result in which only less than ten percent of women disagreed to men’s justification of physical violence the present finding was lower [12].

Overall, the study showed noticeably higher prevalence of utilization of the maternal health care services among the study population than the national survey result of the 2011 EDHS report [6]. Most of the women in this study had ANC visits compared to the 2011 EDHS report (95.2% vs 34% at least once, and 35.2% vs 19% four or more times), and most of the women delivered with the assistance of a skilled health care provider (85.7% vs 12%). This difference could be attributed to the study setting in which urban residents use the service more than rural [7, 29]. The finding of this study was also relatively higher than the finding of a recent study done in Addis Ababa [30], which reported only 24% utilization of four or more ANC. This could probably be because the participants of the latter study were pregnant and may not finished their ANC visits. However, this study showed that nearly one in twenty of the women had no ANC during their pregnancy implying that substantial proportion of the women missed the opportunity of benefitting from essential maternal health care services.

The study found inconsistent relationships between the four forms of IPV and utilization of maternal health care services during pregnancy. The findings confirmed some of the study hypothesis in that women who ever experienced physical IPV in their relationship were less likely to use ANC four or more times, test for HIV, deliver with skilled birth attendant, and deliver in a health facility. This was consistent with the findings of Goo & Harlow, Makayoto et al., and Rahman et al. [23–25].

Other than these, the study also found a significant association to exist between emotional IPV and likelihood of having 1st ANC within three months of gestational age. Women who reported ever been experienced emotional IPV in their relationship were less likely to have their 1st ANC within the 1^st^ trimester of their pregnancy, which supports the report of a study in Nigeria [19]. Similarly, the study found that women who have been sexually abused in their relationship were less likely to use ANC four or more times.

Women who have experienced partner control in their relationship were less likely to use ANC four or more times and the finding was in line with the report from the EDHS 2011 data, which reported that utilization of ANC is higher among more autonomous women in Ethiopia [7] and attributed to low women’s confidence in seeking healthcare service due to male partner’s control.

### Strength and limitation of the study

This study generated evidences and filled an important research gap and broadened our understanding in the association between IPV and maternal health care services utilization. In addition, the study was community based and reliability of the data was maintained by prior training for data collectors and regular supervision by principal investigator and using pretest questionnaire.

However, the study has some limitations. The sample size was small and the study was a cross-sectional study that does not allow exploring causality, it was not easy to measure the temporal relationship since both exposure and outcome variables were collected simultaneously. The nature of the subject, associated with stigmatization, could lend the possibility of under reporting of IPV.

## Conclusions

IPV is prevalent among couples in Addis Ababa, Ethiopia where three out of four women reported having experienced one or another type of IPV in their current relationship. And all types of IPV showed significant association with poor utilization of one or more maternal health care services. Thus efforts to sustain the recent success in maternal health in Addis Ababa, Ethiopia and further improvement should give due consideration to IPV. Further work is needed to further understand the association between IPV and maternal health care service utilization.

## Additional files

Additional file 1: Questionnaire for men (DOCX 59 kb)
Additional file 2: Questionnaire for women (DOCX 60 kb)

## Abbreviations

ANC: Antenatal care
AOR: Adjusted odds ratio
EDHS: ETHIOPIAN demographic and health survey
HEW: Health extension workers
IPV: Intimate partner violence
MDG: Millennium development goals
MTCT: Mother-to-child transmission
PMTCT: Prevention of mother-to-child transmission of HIV
SDG: Sustainable development goals
