# Supplementary material for: Intimate partner violence and utilization of maternal health care services in Addis Ababa, Ethiopia
Source: BMC Health Serv Res. 2017 Mar 7;17:178. doi: 10.1186/s12913-017-2121-7 (PMC5341201; doi:10.1186/s12913-017-2121-7)
Supplement: Additional file 1: — Questionnaire for men (DOCX 59 kb) [file 12913_2017_2121_MOESM1_ESM.docx]

*Questionnaire ID Number | | | |*

**School of Public Health and Primary Care**

**The Chinese University of Hong Kong**

Men’s involvement in Maternal and Child Health

**Questionnaire for MEN**

***Introduction***

Dear Sir:

On behalf of the research team, I would like to thank you for your participation in our study. Your participation will help us to understand men’s involvement in maternal and child health. I would like to reassure you that your answer to the questions is ***COMPLETELY CONFIDENTIAL***. The information you provide will be coded and will never be disclosed to anyone including your partner. Your complete honesty is crucial. Please try to answer all questions. It will only take about 30 minutes of your time.

| ***To be filled by data collector:*** |
| --- |
| Informed consent has been signed ☐ Yes ☐ No |
| ☐ Both partners completed ☐ Only one partner completed |
| Data collector’s name: Signature: Date: . |
|  |
| ***Checked by investigator:*** |
| Signature: _ Date: . |
|  |

**Socio-demographic Characteristics: SDC**

| **SDC:** Let us start with some basic information about you. Please answer the following questions accordingly. | |
| --- | --- |
| SDC1 | In what month and year were you born? Year: ; Month: ______________ |
| SDC2 | What is the highest level of education you completed?  1☐ Never attended school 2☐Primary (1-8) 3☐Secondary school (9-12) 4☐Some College/University 5☐College/University graduate |
| SDC3 | Which ethnicity do you belong to? 1☐ Amhara 2☐ Oromo 3☐ Somali 4☐ Tigray 5☐ Other specify: |
| SDC4 | Which religion do you belong to? 1☐ Orthodox 2☐Muslim 3☐Protestant 4☐Catholic 5☐Other specify: |
| SDC5 | What is your occupation? . |
| SDC6 | What is your monthly personal income, net income after taxes deductions?________ Ethiopian Birr |
| SDC7 | What is the monthly household net income after taxes deductions?_________ Ethiopian Birr |
| SDC8 | Do you own the house you living in or any other house or any land either alone or jointly with someone else? 1☐ Yes 2☐No |
| SDC9 | How long have you been living with your current partner? Years; _______ Months |
| SDC11 | How do you describe your relationship with your current partner? 1☐ Married with certificate 2☐ Religiously married 3☐ Traditionally married 4☐ Living together as if married 5☐ Other specify: |
| SDC12 | How many children do you have with your current partner? . |
| SDC13 | How old is your last child? ________ Months, _________ Days |
| SDC14 | Have you ever been married or lived together with a woman before as if married?  1☐ Yes formerly married 2☐ Yes lived with a woman 3☐ No |
| ***How often do you…*** | |
| SDC15 | Read a newspaper or magazine? 1☐Every day 2☐At least once a week 3☐Less than once a week 4☐Not at all |
| SDC16 | Listen to the radio? 1☐Every day 2☐At least once a week 3☐Less than once a week 4☐Not at all |
| SDC17 | Watch television? 1☐Every day 2☐At least once a week 3☐Less than once a week 4☐Not at all |

**MTCT and PMTCT knowledge, Awareness and Communication - KAC**

| **KAC:** The following questions are about HIV/AIDS please mark the appropriate box to indicate if it is correct or not, or if you’re not sure. | |
| --- | --- |
| KAC1 | Can the virus that causes AIDS be transmitted from an infected mother to her baby?  _1_☐Yes _2_☐No ……… ***Skip to KAC12*** _3_☐I don’t know……… ***Skip to KAC12*** |
| *Can the virus that causes AIDS be transmitted from an infected mother to her baby:* | |
| KAC2 | During pregnancy? _1_☐Yes _2_☐No _3_☐I don’t know |
| KAC3 | During delivery? _1_☐Yes _2_☐No _3_☐I don’t know |
| KAC4 | By breastfeeding? _1_☐Yes _2_☐No _3_☐I don’t know |
| KAC5 | Can mother to child transmission of HIV be prevented? _1_☐Yes _2_☐No  _3_☐I don’t know |
| KAC6 | Are there any special drugs that a doctor or a nurse can give to a woman infected with the AIDS virus to reduce the risk of transmission to the baby? _1_☐Yes _2_☐No _3_☐I don’t know |
| KAC7 | Can delivering baby by cesarean section reduce the chance of transmission of HIV from mother to her child? _1_☐Yes _2_☐No _3_☐I don’t know |
| KAC8 | Can complete avoidance of breastfeeding reduce the chance of transmission of HIV from a mother to her child? _1_☐Yes _2_☐No _3_☐I don’t know |
| KAC9 | Are there any special drugs that a doctor or a nurse can give to a new born baby from a woman infected with the AIDS virus to reduce the risk of transmission to the baby? _1_☐Yes _2_☐No _3_☐I don’t know |
| KAC10 | Have you ever heard of a program called prevention of mother to child transmission of HIV?  _1_☐Yes _2_☐No _3_☐I don’t remember |
| KAC11 | Do you know that prevention of mother to child transmission of HIV services are offered in some health facilities? _1_☐Yes _2_☐No _3_☐I don’t know |
| KAC12 | Have you ever discussed with your partner about mother to child transmission of HIV or ways to prevent it? _1_☐Yes _2_☐No _3_☐I don’t remember |
| KAC13 | If you discussed, who initiated it? _1_☐You _2_☐Your partner _3_☐I don’t remember |

**Attitudes towards PMTCT of HIV interventions – ATP**

| **ATP:** For each of the following statements, please choose the answer that best reflects your personal feelings. On the scale 1 to 5 [**1 = Strongly Disagree 2 = Disagree 3 = Undecided 4 = Agree 5 = Strongly Agree]** mark your level of agreement to the following statements. | |
| --- | --- |
| ATP1 | Pregnant women should be tested for HIV 1☐ 2☐ 3☐ 4☐ 5☐ |
| ATP2 | Pregnant women with HIV should be referred to institutions where they can be monitored.  1☐ 2☐ 3☐ 4☐ 5☐ |
| ATP3 | Women with HIV should use antiretroviral drugs during pregnancy. 1☐ 2☐ 3☐ 4☐ 5☐ |
| ATP4 | Pregnant women with HIV must deliver with skilled personnel. 1☐ 2☐ 3☐ 4☐ 5☐ |
| ATP5 | Pregnant women with HIV should not deliver at home or at traditional birth attendants.  1☐ 2☐ 3☐ 4☐ 5☐ |
| ATP6 | A woman with HIV may not breastfeed her child if there is risk of infection. 1☐ 2☐ 3☐ 4☐ 5☐ |

**Level of involvement in PMTCT - LIP**

| **LIP:** Please answer the following questions accordingly by marking the box. | |
| --- | --- |
| LIP1 | Have you ever initiated a discussion about mother to child transmission of HIV with your partner? 1☐ Yes 2☐ No 3☐ I don’t remember |
| LIP2 | Have you ever requested your partner to be tested for HIV during pregnancy? 1☐ Yes 2☐ No 3☐ My partner is HIV+ prior to pregnancy |
| LIP3 | When your partner had antenatal care follow-up, have you ever asked her what information or services she got in the clinic? 1☐ Yes 2☐ No 3☐ My partner had no antenatal follow-up |
| LIP4 | Have you ever reminded your partner of her antenatal care follow up (schedule)?  1☐ Yes 2☐ No 3☐ I don’t remember |
| LIP5 | Did you pay for medical/transport expenses of your partner in the ANC follow up of this pregnancy? 1☐ Yes 2☐ No 3☐ I don’t remember |
| LIP6 | Did you accompany your partner to antenatal care clinic at least once during her last pregnancy? 1☐ Yes 2☐ No 3☐ I don’t remember |
| LIP7 | If you accompanied your partner, did you enter in to the antenatal care room together with your partner? 1☐ Yes 2☐ No 3☐ I don’t remember |
| LIP8 | Have you been counseled and tested for HIV during your partner’s last pregnancy? 1☐ Yes 2☐ No 3☐ I tested HIV+ prior to her pregnancy |
| LIP9 | If you have been tested, were you counseled and tested together with your partner?  1☐ Yes 2☐ No 3☐ My partner was HIV+ pre-pregnancy |
| LIP10 | Assuming you were to have an HIV test now, will you reveal your result to your partner if you test positive for HIV? 1☐ Yes 2☐ No 3☐ I’m not sure 4☐ I am HIV+ |
| LIP11 | If your partner tested for HIV and tested positive, will you accept that she and the newborn take antiretroviral drugs to prevent transmission of the virus from mother to the baby? 1☐ Yes 2☐ No 3☐ I’m not sure |
| LIP12 | If your partner tested positive for HIV, would you be confident to help in the newborn’s medical follow up? 1☐ Yes 2☐ No 3☐ I’m not sure |
| LIP13 | If you and your partner tested for HIV and your partner tested positive while you tested negative, would you continue your relationship with her? 1☐ Yes 2☐ No 3☐ I’m not sure |
| LIP14 | If you and your partner tested for HIV and your partner tested negative while you tested positive, would you be confident to use condom consistently to prevent transmission to her and thus to the child? 1☐ Yes 2☐ No 3☐ I’m not sure |

**Experience of ANC attendance - EAA**

| **PAA:** The following questions are concerning your support during your partner’s last pregnancy. Please answer the questions accordingly. | |
| --- | --- |
| EAA1 | Have you ever been asked by your partner to go to ANC clinic with her during her last pregnancy?  _1_☐ Yes _2_☐ No ……… ***Skip to EAA4*** _3_☐ I don’t remember ……… ***Skip to EAA4*** |
| EAA2 | If you were asked, did you accept? _1_☐ Yes ……… ***Skip to OPA1*** _2_☐ No _3_☐ I don’t remember |
| EAA3 | If you did not accept, what was the reason? [You can mark more than 1 option] |
| 1☐ I have no time 2☐ I do not have anything to do there 3☐ It is not me who is pregnant 4☐ I did not wish to answer 5☐ Other specify: | |
| EAA4 | If you have never been asked, would you accept if she did ask you to go to ANC clinic with her?  _1_☐ Yes _2_☐ No _3_☐ I don’t Know |
| EAA5 | If you say ‘no’, why you wouldn’t accept to go with her? [You can mark more than 1 option] |
| 1☐ I have no time 2☐ I do not have anything to do there 3☐ It is not me who is pregnant 4☐ I don't have to answer 5☐ Other specify: | |

**Opinion or belief about partner’s attendance at ANC - OPA**

| **OPA:** Now we want to know what you think about men’s attendance to antenatal care clinic with women. Based on your personal opinions, please share your feelings about it. | |
| --- | --- |
| OPA1 | Is it good for a man to go to antenatal clinic with his partner?  _1_☐ Yes _2_☐ No …. ***Skip to* OPA3** _3_☐ I don’t Know…. ***Skip to* OPA4** |
| OPA2 | If you say ‘yes’, why is it good for a man to go to antenatal clinic? [You can mark more than 1 option] |
| 1☐ Both can have HIV testing and know status together 2☐ It increases his knowledge of antenatal activities 3☐ In case of infection they can be treated together 4☐ It shows real love and faithfulness for each other  5☐ It makes her happy and feel she is supported 6☐ The man will benefit from first hand information  7☐Other specify: | |
| OPA3 | If you say ‘no’, why is it not good for a man to go to antenatal clinic? [You can mark more than one option] |
| 1☐ Men do not have time to come 2☐ Pregnancy is a woman’s affair 3☐ It is not our culture 4☐ The woman may be ashamed and uncomfortable 5☐ The health workers may not welcome him  6☐ His other partner will be jealous 7☐Other specify: | |
| OPA4 | What do most men in your area think about men who go to ANC clinic with their partners? [You can mark more than one options] |
| 1☐ It is normal 2☐ It is not normal 3☐ The man is jealous and overprotective 4☐ It is an act of responsibility and true love 5☐ It is a sign of weakness in the man 6☐Other specify: | |

**Attitudes towards “gender-equitable” norms - GEN**

| **GEN:** Please rate your level of agreement to the following statements. Mark only one box for each that best reflects your feelings. | | _1_**Agree** | _2_**Partially Agree** | _3_**Don’t Agree** |
| --- | --- | --- | --- | --- |
| GEN1 | There are times when a woman deserves to be beaten. | ☐ | ☐ | ☐ |
| GEN2 | A woman should tolerate violence to keep her family together. | ☐ | ☐ | ☐ |
| GEN3 | It is all right for a man to beat his wife if she is unfaithful. | ☐ | ☐ | ☐ |
| GEN4 | A man can hit his wife if she won’t have sex with him. | ☐ | ☐ | ☐ |
| GEN5 | If someone insults a man, he should defend his reputation with force if he has to. | ☐ | ☐ | ☐ |
| GEN6 | A man using violence against his wife is a private matter that shouldn’t be discussed outside the couple. | ☐ | ☐ | ☐ |
| GEN7 | It is the man who decides what type of sex to have. | ☐ | ☐ | ☐ |
| GEN8 | Men are always ready to have sex. | ☐ | ☐ | ☐ |
| GEN9 | Men need sex more than women do. | ☐ | ☐ | ☐ |
| GEN10 | A man needs other women even if things with his wife are fine. | ☐ | ☐ | ☐ |
| GEN11 | You don’t talk about sex, you just do it. | ☐ | ☐ | ☐ |
| GEN12 | It disgusts me when I see a man acting like a woman. | ☐ | ☐ | ☐ |
| GEN13 | A woman should not initiate sex. | ☐ | ☐ | ☐ |
| GEN14 | A woman who has sex before she marries does not deserve respect. | ☐ | ☐ | ☐ |
| GEN15 | Women who carry condoms on them are easy. | ☐ | ☐ | ☐ |
| GEN16 | Men should be outraged if their wives ask them to use a condom. | ☐ | ☐ | ☐ |
| GEN17 | It is a woman’s responsibility to avoid getting pregnant. | ☐ | ☐ | ☐ |
| GEN18 | Only when a woman has a child is she a real woman. | ☐ | ☐ | ☐ |
| GEN19 | A real man produces a male child. | ☐ | ☐ | ☐ |
| GEN20 | Changing dippers, giving a bath, and feeding kids is the mother’s responsibility. | ☐ | ☐ | ☐ |
| GEN21 | A woman’s role is taking care of her home and family. | ☐ | ☐ | ☐ |
| GEN22 | The husband should decide to buy the major household items. | ☐ | ☐ | ☐ |
| GEN23 | A man should have the final word about decisions in his home. | ☐ | ☐ | ☐ |
| GEN24 | A woman should obey her husband in all things. | ☐ | ☐ | ☐ |

**Men’s attitude toward their Masculinity - MRA**

| **MRA:** Please rate your level of agreement to the following statements. Mark  only one box for each that best reflects your feelings. | | _1_**Agree** | _2_**Partially Agree** | _3_**Don’t Agree** |
| --- | --- | --- | --- | --- |
| MRA1 | It is essential for a guy to get respect from others | ☐ | ☐ | ☐ |
| MRA2 | A man always deserves the respect of his wife and children | ☐ | ☐ | ☐ |
| MRA3 | I admire a guy who is totally sure of himself | ☐ | ☐ | ☐ |
| MRA4 | A guy will lose respect if he talks about his problems | ☐ | ☐ | ☐ |
| MRA5 | A young man should be physically tough, even if he’s not big | ☐ | ☐ | ☐ |

**Health system factors - HSF**

| **HSF:** Please rate your level of agreement to the following statements. Mark  only one box for each that best reflects your feelings. **1 = Strongly Agree 2 = Agree 3 = Undecided 4 = Disagree 5 = Strongly Disagree** | | | | | | |
| --- | --- | --- | --- | --- | --- | --- |
| HSF1 | ANC/PMTCT clinics should be opened on weekends and evening for men to attend with their partner. | _1_☐ | _2_☐ | _3_☐ | _4_☐ | _5_☐ |
| HSF2 | Long distance to the health facility is an obstacle to attend ANC/PMTCT clinic with your partner. | _1_☐ | _2_☐ | _3_☐ | _4_☐ | _5_☐ |
| HSF3 | Couple counseling and testing for HIV should be conducted in the community rather than ANC/PMTCT clinics. | _1_☐ | _2_☐ | _3_☐ | _4_☐ | _5_☐ |
| HSF4 | There should be separate waiting areas for men and women visiting ANC/PMTCT. | _1_☐ | _2_☐ | _3_☐ | _4_☐ | _5_☐ |
| HSF5 | There should be a different exit after HIV testing to avoid being identified by the crowd waiting for service. | _1_☐ | _2_☐ | _3_☐ | _4_☐ | _5_☐ |
| HSF6 | A long waiting time at the clinic during ANC visit is an obstacle to attend ANC/PMTCT with your partner. | _1_☐ | _2_☐ | _3_☐ | _4_☐ | _5_☐ |
| HSF7 | I have noticed health facilities inviting/promoting male-partners’ involvement in ANC/PMTCT through mass media. | _1_☐ | _2_☐ | _3_☐ | _4_☐ | _5_☐ |
| HSF8 | I have seen a signboard with picture or message promoting male involvement in PMTCT at gates or in premise of any health facilities or other areas in the city. | _1_☐ | _2_☐ | _3_☐ | _4_☐ | _5_☐ |
| HSF9 | When your partner had ANC/PMTCT follow up, have you been invited verbally or in writing for your attendance by the health facility. | _1_☐ | _2_☐ | _3_☐ | _4_☐ | _5_☐ |
| HSF10 | From what you have observed or heard service providers do not request men in waiting area to enter in to ANC together with their partner. | _1_☐ | _2_☐ | _3_☐ | _4_☐ | _5_☐ |
| HSF11 | Facilities do give men medical certificate of ANC attendance if required by employee. | _1_☐ | _2_☐ | _3_☐ | _4_☐ | _5_☐ |
| HSF12 | Rudeness and rough handling of the pregnant women by the service providers in the clinics was an obstacle for men to attend ANC/PMTCT clinics. | _1_☐ | _2_☐ | _3_☐ | _4_☐ | _5_☐ |
| HSF13 | Health service providers at the ANC/PMTCT clinics keep HIV test results a secret. | _1_☐ | _2_☐ | _3_☐ | _4_☐ | _5_☐ |

**Control over partner - COP**

| COP1: On a scale of 1-4 how controlling you are over your partner:  _1_☐ Very Controlling _2_☐ Controlling _3_☐ Slightly Controlling _4_☐ Not Controlling |
| --- |

**Couple’s Relationship Quality - CRQ**

| **CRQ:** For each of the following statements, please choose the level of agreement that best reflects your personal feeling about your relationship with your current partner.  On the scale 1 to 5 **[*1 = Strongly Disagree 2 = Disagree 3 = Undecided 4 = Agree 5 = Strongly Agree*]** mark your level of agreement to the following statements. | | | | | | |
| --- | --- | --- | --- | --- | --- | --- |
| CRQ1 | I expect my love for my current partner to last for the rest of my life. | _1_☐ | _2_☐ | _3_☐ | _4_☐ | _5_☐ |
| CRQ2 | I view my relationship with my current partner as permanent. | _1_☐ | _2_☐ | _3_☐ | _4_☐ | _5_☐ |
| CRQ3 | I am committed to maintaining my relationship with my current partner. | _1_☐ | _2_☐ | _3_☐ | _4_☐ | _5_☐ |
| CRQ4 | I have confidence in the stability of my relationship with my current partner. | _1_☐ | _2_☐ | _3_☐ | _4_☐ | _5_☐ |
| CRQ5 | My partner is perfectly honest and truthful with me. | _1_☐ | _2_☐ | _3_☐ | _4_☐ | _5_☐ |
| CRQ6 | I feel I can trust my partner completely. | _1_☐ | _2_☐ | _3_☐ | _4_☐ | _5_☐ |
| CRQ7 | My partner is truly sincere in her promises. | _1_☐ | _2_☐ | _3_☐ | _4_☐ | _5_☐ |
| CRQ8 | My partner treats me fairly and justly. | _1_☐ | _2_☐ | _3_☐ | _4_☐ | _5_☐ |
| CRQ9 | I feel that my partner can be counted on to help me. | _1_☐ | _2_☐ | _3_☐ | _4_☐ | _5_☐ |
| CRQ10 | My partner and I try to discuss problems. | _1_☐ | _2_☐ | _3_☐ | _4_☐ | _5_☐ |
| CRQ11 | My partner and I express our feelings to each other. | _1_☐ | _2_☐ | _3_☐ | _4_☐ | _5_☐ |
| CRQ12 | We suggest possible solutions and compromises. | _1_☐ | _2_☐ | _3_☐ | _4_☐ | _5_☐ |
| CRQ13 | We threaten each other with negative consequences. | _1_☐ | _2_☐ | _3_☐ | _4_☐ | _5_☐ |
| CRQ14 | I call my partner names, swear at her or attack her character. | _1_☐ | _2_☐ | _3_☐ | _4_☐ | _5_☐ |
| CRQ15 | My partner calls me names, swears at me or attacks my character. | _1_☐ | _2_☐ | _3_☐ | _4_☐ | _5_☐ |

**Couples Relationship Satisfaction - CRS**

| **CRS:** Now we want to know your personal satisfaction in your relationship with your current partner. Based on your personal opinions, please share your feelings about it. | |
| --- | --- |
| CRS1 | How well does your partner meet your needs?  _1_☐ _2_☐ _3_☐ _4_☐ _5_☐  Poorly Average Extremely well |
| CRS2 | In general, how satisfied are you with your relationship?  _1_☐ _2_☐ _3_☐ _4_☐ _5_☐  Unsatisfied Average Extremely satisfied |
| CRS3 | How good is your relationship compared to most?  _1_☐ _2_☐ _3_☐ _4_☐ _5_☐  Poor Average Excellent |
| CRS4 | How often do you wish you hadn’t gotten into this relationship?  _5_☐ _4_☐ _3_☐ _2_☐ _1_☐  Never Average Very often |
| CRS5 | To what extent has your relationship met your original expectations?  _1_☐ _2_☐ _3_☐ _4_☐ _5_☐  Hardly at all Average Completely |
| CRS6 | How much do you love your partner?  _1_☐ _2_☐ _3_☐ _4_☐ _5_☐  Not much Average Very much |
| CRS7 | How many problems are there in your relationship?  _5_☐ _4_☐ _3_☐ _2_☐ _1_☐  Very few Average Very many |

**Religiosity - REL**

| **REL:** The following section contains 3 statements about religious belief or experience. Please mark the extent to which each statement is true or not true for you. | |
| --- | --- |
| REL1 | In my life, I experience the presence of the Divine (i.e., God).  _1_☐Definitely not true _2_☐ Tends not to be true _3_☐ Unsure _4_☐ Tends to be true _5_☐ Definitely true of me |
| REL2 | My religious beliefs are what really lie behind my whole approach to life.  _1_☐Definitely not true _2_☐ Tends not to be true _3_☐ Unsure _4_☐ Tends to be true _5_☐ Definitely true of me |
| REL3 | I try hard to carry my religion over into all other dealings in life.  _1_☐Definitely not true _2_☐ Tends not to be true _3_☐ Unsure _4_☐ Tends to be true _5_☐ Definitely true of me |

**Sexual Relationship Power - SRP**

| **SRP:** Please rate your level of agreement to the following statements. Mark the one box for each statement that best reflects your personal feelings. ***1=Strongly agree, 2=Agree, 3=Disagree, 4=Strongly disagree*** | | | | | |
| --- | --- | --- | --- | --- | --- |
| SRP1 | Under no circumstances would I ever leave my partner. | _1_☐ | _2_☐ | _3_☐ | _4_☐ |
| SRP2 | If my partner were to leave me, I would be in serious trouble. | _1_☐ | _2_☐ | _3_☐ | _4_☐ |
| SRP3 | If things were really bad with my partner, I would leave the relationship. | _1_☐ | _2_☐ | _3_☐ | _4_☐ |
| SRP4 | If my partner failed to meet my needs, I could easily find another partner. | _1_☐ | _2_☐ | _3_☐ | _4_☐ |
| SRP5 | My partner and I sit down and discuss important matters together. | _1_☐ | _2_☐ | _3_☐ | _4_☐ |
| SRP6 | My partner shows that she care about me. | _1_☐ | _2_☐ | _3_☐ | _4_☐ |
| SRP7 | If I suspect my partner is having an affair, I would talk with my partner. | _1_☐ | _2_☐ | _3_☐ | _4_☐ |
| SRP8 | I would consult with my advisors/friends if my partner was behaving badly. | _1_☐ | _2_☐ | _3_☐ | _4_☐ |
| SRP9 | When I need my partner's assistance, she is always there to help me. | _1_☐ | _2_☐ | _3_☐ | _4_☐ |
| SRP10 | I initiate sex with my partner when I want to have sex. | _1_☐ | _2_☐ | _3_☐ | _4_☐ |
| SRP11 | I am able to buy expensive items without my partner’s approval. | _1_☐ | _2_☐ | _3_☐ | _4_☐ |
| SRP12 | I have my own money to buy things I want. | _1_☐ | _2_☐ | _3_☐ | _4_☐ |
| SRP13 | My partner punishes me when she is angry with me. | _1_☐ | _2_☐ | _3_☐ | _4_☐ |
| SRP14 | When I disagree with my partner's relatives, my partner chooses their side over mine. | _1_☐ | _2_☐ | _3_☐ | _4_☐ |
| SRP15 | My partner is probably having sex with someone else. | _1_☐ | _2_☐ | _3_☐ | _4_☐ |
| SRP16 | If my partner was really angry with me, she might beat me. | _1_☐ | _2_☐ | _3_☐ | _4_☐ |

**HIV Risk and risk perception - RRP**

| **RRP:** The following questions are about your HIV sero-status and perception on the likelihood that you are at risk of getting HIV. Please answer accordingly. *YOUR RESPONSE IS CONFIDENTIAL!* | |
| --- | --- |
| RRP1 | On the scale 1 to 5, 1 being VERY LIKELY and 5 VERY UNLIKELY; please rate how likely you are at risk of getting HIV infection. _1_☐ _2_☐ _3_☐ _4_☐ _5_☐  _6_☐ *I’m HIV positive ….* ***Skip to RRP4*** |
| RRP2 | Do you think that your current sexual behaviour puts you at risk of HIV infection?  _1_☐ Yes _2_☐ No _3_☐ I don’t know |
| RRP3 | Do you think that your partner’s current sexual behaviour puts you at risk of HIV infection?  _1_☐ Yes _2_☐ No _3_☐ I don’t know |
| RRP4 | Have you ever had sexual intercourse with anyone other than your partner in the last twelve months? _1_☐ Yes _2_☐ No |
| RRP5 | How often do you use condom during your non-partner sexual intercourse?  _1_☐ Never _2_☐ Sometimes _3_☐ Usually but not always _4_☐ Always |
| RRP6 | How often do you use condom during sexual intercourse with your current partner?  _1_☐ Never _2_☐ Sometimes _3_☐ Usually but not always _4_☐ Always |

Finally, we would like to include your partner in the study as well. Can you invite her to participate? ☐ Yes ☐ No, why would you not invite him

**Thank you for your participation!**

Please return the questionnaire according to the instructions provided.
