# Supplementary material for: Intimate partner violence and utilization of maternal health care services in Addis Ababa, Ethiopia
Source: BMC Health Serv Res. 2017 Mar 7;17:178. doi: 10.1186/s12913-017-2121-7 (PMC5341201; doi:10.1186/s12913-017-2121-7)
Supplement: Additional file 2: — Questionnaire for women (DOCX 60 kb) [file 12913_2017_2121_MOESM2_ESM.docx]

*Questionnaire ID Number | | | |*

**School of Public Health and Primary Care**

**The Chinese University of Hong Kong**

Men’s involvement in Maternal and Child Health

**Questionnaire for WOMEN**

***Introduction***

Dear Madam:

On behalf of the research team, I would like to thank you for your participation in our study. Your participation will help us to understand men’s involvement in maternal and child health. I would like to reassure you that your answer to the questions is ***COMPLETELY CONFIDENTIAL***. The information you provide will be coded and will never be disclosed to anyone including your partner. Your complete honesty is crucial. Please try to answer all questions. It will only take about 30 minutes of your time.

| ***To be filled by data collector:*** |
| --- |
| Informed consent has been signed ☐ Yes ☐ No |
| ☐ Both partners completed ☐ Only one partner completed |
| Data collector’s name: Signature: Date: . |
|  |
| ***Checked by investigator:*** |
| Signature: Date: . |
|  |

**Socio-demographic Characteristics: SDC**

| **SDC:** Let us start with some basic information about you. Please answer the following questions accordingly. | |
| --- | --- |
| SDC1 | In what month and year were you born? Month: Year: . |
| SDC2 | What is the highest level of education you completed? 1☐ Never attended school 2☐Some primary (1-8) 3☐Secondary school (9-12) 4☐Some College/University 5☐College/University graduate |
| SDC3 | Which ethnicity do you belong to? 1☐ Amhara 2☐ Oromo 3☐ Somali 4☐ Tigray 5☐ Other specify: |
| SDC4 | Which religion do you belong to? 1☐ Orthodox 2☐Muslim 3☐Protestant 4☐Catholic 5☐Other specify: |
| SDC5 | What is your occupation? . |
| SDC6 | What is your monthly personal income, net income after taxes deductions?________ Ethiopian Birr |
| SDC7 | What is the monthly household net income after taxes deductions?_________ Ethiopian Birr |
| SDC8 | Do you own the house you living in or any other house or any land either alone or jointly with someone else? 1☐ Yes 2☐No |
| SDC9 | How long have you been living with your current partner? Years. |
| SDC10 | Does your partner have partner/s other than you? 1☐No 2☐Yes 3☐ If your answer is yes, how many? _____________ |
| SDC11 | How do you describe your relationship with your current partner? 1☐ Married with certificate 2☐ Religiously married 3☐ Traditionally married 4☐ Living together as if married 5☐ Other specify: |
| SDC12 | How many children do you have with your current partner? . |
| SDC13 | How old is your last child? ________ Months, ________ days |
| SDC14 | Have you ever been married or lived together with a man as if married?  1☐ Yes formerly married 2☐ Yes lived with a man 3☐ No |
| ***How often do you…*** | |
| SDC15 | Read a newspaper or magazine? 1☐Every day 2☐At least once a week 3☐Less than once a week 4☐Not at all |
| SDC16 | Listen to the radio? 1☐Every day 2☐At least once a week 3☐Less than once a week 4☐Not at all |
| SDC17 | Watch television? 1☐Every day 2☐At least once a week 3☐Less than once a week 4☐Not at all |

**MTCT and PMTCT knowledge, Awareness and Communication - KAC**

| **KAC:** The following questions are about HIV/AIDS please mark the appropriate box to indicate if it is correct or not, or if you’re not sure. | |
| --- | --- |
| KAC1 | Can the virus that causes AIDS be transmitted from an infected mother to her baby?  _1_☐Yes _2_☐No ……… ***Skip to KAC12*** _3_☐I don’t know……… ***Skip to KAC12*** |
| *Can the virus that causes AIDS be transmitted from an infected mother to her baby:* | |
| KAC2 | During pregnancy? _1_☐Yes _2_☐No _3_☐I don’t know |
| KAC3 | During delivery? _1_☐Yes _2_☐No _3_☐I don’t know |
| KAC4 | By breastfeeding? _1_☐Yes _2_☐No _3_☐I don’t know |
| KAC5 | Can mother to child transmission of HIV be prevented? _1_☐Yes _2_☐No  _3_☐I don’t know |
| KAC6 | Are there any special drugs that a doctor or a nurse can give to a woman infected with the AIDS virus to reduce the risk of transmission to the baby? _1_☐Yes _2_☐No _3_☐I don’t know |
| KAC7 | Can delivering baby by cesarean section reduce the chance of transmission of HIV from mother to her child? _1_☐Yes _2_☐No _3_☐I don’t know |
| KAC8 | Can complete avoidance of breastfeeding reduce the chance of transmission of HIV from a mother to her child? _1_☐Yes _2_☐No _3_☐I don’t know |
| KAC9 | Are there any special drugs that a doctor or a nurse can give to a new born baby from a woman infected with the AIDS virus to reduce the risk of transmission to the baby? _1_☐Yes _2_☐No _3_☐I don’t know |
| KAC10 | Have you ever heard of a program called prevention of mother to child transmission of HIV?  _1_☐Yes _2_☐No _3_☐I don’t remember |
| KAC11 | Do you know that prevention of mother to child transmission of HIV services are offered in some health facilities? _1_☐Yes _2_☐No _3_☐I’m not sure |
| KAC12 | Have you ever discussed with your partner about mother to child transmission of HIV or ways to prevent it? _1_☐Yes _2_☐No _3_☐I don’t remember |
| KAC13 | If you discussed, who initiated it? _1_☐You _2_☐Your partner _3_☐I don’t remember |

**Access to ANC/PMTCT services - ATA**

| **ATA:** The following questions refer to your experience in using some of health services during your last pregnancy. Please answer the questions accordingly. | |
| --- | --- |
| ATA1 | Have you attended antenatal clinic during your last pregnancy? _1_☐Yes  _2_☐No ….. ***Skip to* ATA16** |
| ATA2 | How many months pregnant were you when you first received antenatal care for your last pregnancy? Months |
| ATA3 | How many times did you receive antenatal care during your last pregnancy? _________ |
| ***During any of the antenatal visits for your last pregnancy, did anyone talk to you about:*** | |
| ATA4 | *babies getting the AIDS virus from their mother? _1_☐Yes _2_☐No _3_☐Don’t remember* |
| ATA5 | *things that you can do to prevent getting the AIDS virus? _1_☐Yes _2_☐No _3_☐Don’t remember* |
| ATA6 | *getting tested for the AIDS virus? _1_☐Yes _2_☐No _3_☐Don’t remember* |
| ATA7 | Were you offered a test for the AIDS virus as part of your antenatal care? _1_☐Yes _2_☐No |
| ATA8 | Were you tested for the AIDS virus as part of your antenatal care? _1_☐Yes  _2_☐No ….***Skip to* ATA14** |
| ATA9 | How many months pregnant were you when you tested for HIV? _______Months |
| ATA10 | Did you get the result of the test? _1_☐Yes _2_☐No…. ***Skip to* ATA14** |
| ATA11 | What was the test result? _1_☐Positive _2_☐Negative ……. ***Skip to* ATA18** |
| ATA12 | Were you offered Antiretroviral drug to prevent transmission of the AIDS virus to the baby?  _1_☐Yes _2_☐No…. ***Skip to* ATA14** |
| ATA13 | Did you get the Antiretroviral drug to prevent transmission of the AIDS virus to the baby?_1_☐Yes _2_☐No |
| ATA14 | Have you tested the baby for the virus? _1_☐Yes _2_☐No |
| ATA15 | What was the test result? _1_☐Positive _2_☐Negative _3_☐ I didn’t receive the result  …. ***Skip to* ATA18** |
| ATA16 | Have you ever been tested to see if you have the AIDS virus? _1_☐Yes _2_☐No…. ***Skip to* ATA19** |
| ATA17 | What was the test result? _1_☐Positive _2_☐Negative |
| ATA18 | The last time you were tested for HIV, did you share the result with your partner? _1_☐Yes _2_☐No |
| ATA19 | Who assisted with the delivery of your last baby? 1☐Doctor 2☐ Nurse/Midwife  3☐ HEW 4☐ Traditional birth attendant 5☐ Other specify: . |
| ATA20 | Where did you deliver your last baby? 1☐Hospital 2☐ Clinic 3☐ Home 4☐ Other specify: |
| ATA21 | What do you feed your baby? 1☐ Breast milk 2☐ Formula 3☐ Both 4☐ Other specify: |

**Attitudes towards PMTCT of HIV interventions – ATP**

| **ATP:** For each of the following statements, please choose the answer that best reflects your personal feelings. On the scale 1 to 5 [**1 = Strongly Disagree 2 = Disagree 3 = Undecided 4 = Agree 5 = Strongly Agree]** mark your level of agreement to the following statements. | |
| --- | --- |
| ATP1 | Pregnant women should be tested for HIV 1☐ 2☐ 3☐ 4☐ 5☐ |
| ATP2 | Pregnant women with HIV should be referred to institutions where they can be monitored.  1☐ 2☐ 3☐ 4☐ 5☐ |
| ATP3 | Women with HIV should use antiretroviral drugs during pregnancy. 1☐ 2☐ 3☐ 4☐ 5☐ |
| ATP4 | Pregnant women with HIV must deliver with skilled personnel. 1☐ 2☐ 3☐ 4☐ 5☐ |
| ATP5 | Pregnant women with HIV should not deliver at home or at traditional birth attendants.  1☐ 2☐ 3☐ 4☐ 5☐ |
| ATP6 | A woman with HIV may not breastfeed her child if there is risk of infection. 1☐ 2☐ 3☐ 4☐ 5☐ |

**Women’s experience of partner’s attendance at ANC - EPA**

| **EPA:** The following questions are concerning your partner’s support during your last pregnancy. Please answer the questions accordingly. | |
| --- | --- |
| EPA1 | Has your partner attended antenatal care clinic with you during your last pregnancy?  _1_☐Yes _2_☐No…. ***Skip to* EPA4** |
| EPA2 | How many times did he attend antenatal care clinic with you? _1_☐Once _2_☐ Sometimes _3_☐All your ANC visits |
| EPA3 | Did he enter to the antenatal care clinic room with you? _1_☐Yes sometimes _2_☐ Yes all the time _3_☐No, Why? ________  …. ***Skip to* OPA1** |
| EPA4 | Have you ever asked your partner to come to antenatal clinic with you? _1_☐Yes _2_☐No…. ***Skip to* EPA6** |
| EPA5 | What did he say? [You can mark more than one options] |
| 1☐ “I have no time” 2☐ “I do not have anything to do there” 3☐ “It is not me who is pregnant” 4☐ He did not wish to answer 5☐ Other specify: | |
| EPA6 | Why have you never asked your partner to come to antenatal clinic? [You can mark more than one options] |
| 1☐ I know he will never go with me 2☐ He does not have time 3☐ He is not around 4☐ He does not have anything to do there 5☐ His presence will make me feel uncomfortable 6☐ Other specify: | |

**Opinion or belief about partner’s attendance at ANC - OPA**

| **OPA:** Now we want to know what you think about men’s attendance to antenatal care clinic with women. Based on your personal opinion, please share your feelings about it. | |
| --- | --- |
| OPA1 | Is it good for a man to come to antenatal clinic with his partner?  _1_☐ Yes _2_☐ No …. ***Skip to* OPA3** _3_☐ I don’t Know…. ***Skip to* OPA4** |
| OPA2 | If you say ‘yes’, why is it good for a man to come to antenatal clinic? [You can mark more than 1 option] |
| 1☐ Both can have HIV testing and know status together 2☐ It increases his knowledge of antenatal activities 3☐ In case of infection they can be treated together 4☐ It shows real love and faithfulness for each other  5☐ It makes her happy and feel she is supported 6☐ The man will benefit from first hand information  7☐Other specify: | |
| OPA3 | If you say ‘no’, why is it not good for a man to come to antenatal clinic? [You can mark more than one option] |
| 1☐ Men do not have time to come 2☐ Pregnancy is a woman’s affair 3☐ It is not our culture 4☐ The woman may be ashamed and uncomfortable 5☐ The health workers may not welcome him  6☐ His other partner will be jealous 7☐Other specify: | |
| OPA4 | What do most women in your area think about men who come to ANC clinic with their partners? [You can mark more than one options] |
| 1☐ It is normal 2☐ It is not normal 3☐ The man is jealous and overprotective 4☐ It is an act of responsibility and true love 5☐ It is a sign of weakness in the man 6☐Other specify: | |

**Household Decision-Making - HDM**

| **HDM:** The following questions are about your household decisions.  *Who in your relationship usually has the final say on the following decisions:* | |
| --- | --- |
| HDM1 | Your own health care? _1_☐Me _2_☐My partner _3_☐Me and my partner together _4_☐Someone else |
| HDM2 | Making large household purchases? _1_☐Me _2_☐My partner _3_☐Me and my partner together _4_☐Someone else |
| HDM3 | Making household purchases for daily needs? _1_☐Me _2_☐My partner _3_☐Me and my partner together _4_☐Someone else |
| HDM4 | Visits to family, friends, or relatives? _1_☐Me _2_☐My partner _3_☐Me and my partner together _4_☐Someone else |

**Partner Intimate Violence - PIV**

| **PIV:** The next questions are about things that happen to many women, and that your current partner may have done to you. *YOUR RESPONSE IS CONFIDENTIAL!*  ***Has your current partner ever....*** | | **A**  (If YES continue with B. If NO skip to next item) | | **B**  Has this happened in the past 12 months? | |
| --- | --- | --- | --- | --- | --- |
|  |  | _1_**Yes** | _2_**No** | _1_**Yes** | _2_**No** |
| PIV1 | Insulted you or made you feel bad about yourself? | ☐ | ☐ | ☐ | ☐ |
| PIV2 | Belittled or humiliated you in front of other people? | ☐ | ☐ | ☐ | ☐ |
| PIV3 | Done things to scare or intimidate you on purpose (e.g. by the way he looked at you, by yelling and smashing things)? | ☐ | ☐ | ☐ | ☐ |
| PIV4 | Threatened to hurt you or someone you care about? | ☐ | ☐ | ☐ | ☐ |
| PIV5 | Slapped you or thrown something at you that could hurt you? | ☐ | ☐ | ☐ | ☐ |
| PIV6 | Pushed you or shoved you or pulled your hair? | ☐ | ☐ | ☐ | ☐ |
| PIV7 | Hit you with his fist or with something else that could hurt you? | ☐ | ☐ | ☐ | ☐ |
| PIV8 | Kicked you, dragged you or beat you up? | ☐ | ☐ | ☐ | ☐ |
| PIV9 | Choked or burnt you on purpose? | ☐ | ☐ | ☐ | ☐ |
| PIV10 | Threatened to use or actually used a gun, knife or other weapon against you? | ☐ | ☐ | ☐ | ☐ |
| PIV11 | Did your partner ever physically force you to have sexual intercourse when you did not want to? | ☐ | ☐ | ☐ | ☐ |
| PIV12 | Did you ever have sexual intercourse you did not want to because you were afraid of what your partner might do? | ☐ | ☐ | ☐ | ☐ |
| PIV13 | Did your partner ever forced you to do something sexual that you found degrading or humiliating? | ☐ | ☐ | ☐ | ☐ |

**Attitude towards Partner Intimate Violence - APIV**

| **APIV:** In your opinion, is a male partner justified in hitting or beating his female partner in the following situations: | |
| --- | --- |
| APIV1 | If she goes out without telling him? _1_☐Yes _2_☐No _3_☐I don’t know |
| APIV2 | If she neglects the children? _1_☐Yes _2_☐No _3_☐I don’t know |
| APIV3 | If she argues with him? _1_☐Yes _2_☐No _3_☐I don’t know |
| APIV4 | If she refuses to have sex with him? _1_☐Yes _2_☐No _3_☐I don’t know |
| APIV5 | If she burns the food? _1_☐Yes _2_☐No _3_☐I don’t know |

**Help in Household Chores - HHC**

| **HHC:** The next questions are about household responsibilities that are often left to women, and that your current partner may/may not have helped you. *YOUR RESPONSE IS CONFIDENTIAL!* | |
| --- | --- |
| HHC1 | Does your current partner help you with household chores like looking after the children, cooking, cleaning the house, and doing other work around the house? _1_☐Yes _2_☐No ***… Skip to PCB1*** |
| HHC2 | Does he help almost _1_☐every day _2_☐at least once a week _3_☐rarely |

**Partner Controlling Behaviours - PCB**

| **PCB:** The next questions are about things that happen to many women, and that your current partner may have done to you. *YOUR RESPONSE IS CONFIDENTIAL!*  ***Has your current partner ever....*** | |
| --- | --- |
| PCB1 | be jealous if you talk with other men? _1_☐Yes _2_☐No |
| PCB2 | accuses you of unfaithfulness?  _1_☐Yes _2_☐No |
| PCB3 | does not permit you to meet your friends? _1_☐Yes _2_☐No |
| PCB4 | tries to limit your contact with family?  _1_☐Yes _2_☐No |
| PCB5 | insists on knowing where you are at all times?  _1_☐Yes _2_☐No |
| PCB6 | doesn’t trust you with money?  _1_☐Yes _2_☐No |

**Couple’s Relationship Quality - CRQ**

| **CRQ:** For each of the following statements, please choose the level of agreement that best reflects your personal feeling about your relationship with your current partner.  On the scale 1 to 5 **[*1 = Strongly Disagree 2 = Disagree 3 = Undecided 4 = Agree 5 = Strongly Agree*]** mark your level of agreement to the following statements. | | | | | | |
| --- | --- | --- | --- | --- | --- | --- |
| CRQ1 | I expect my love for my current partner to last for the rest of my life. | _1_☐ | _2_☐ | _3_☐ | _4_☐ | _5_☐ |
| CRQ2 | I view my relationship with my current partner as permanent. | _1_☐ | _2_☐ | _3_☐ | _4_☐ | _5_☐ |
| CRQ3 | I am committed to maintaining my relationship with my current partner. | _1_☐ | _2_☐ | _3_☐ | _4_☐ | _5_☐ |
| CRQ4 | I have confidence in the stability of my relationship with my current partner. | _1_☐ | _2_☐ | _3_☐ | _4_☐ | _5_☐ |
| CRQ5 | My partner is perfectly honest and truthful with me. | _1_☐ | _2_☐ | _3_☐ | _4_☐ | _5_☐ |
| CRQ6 | I feel I can trust my partner completely. | _1_☐ | _2_☐ | _3_☐ | _4_☐ | _5_☐ |
| CRQ7 | My partner is truly sincere in his promises. | _1_☐ | _2_☐ | _3_☐ | _4_☐ | _5_☐ |
| CRQ8 | My partner treats me fairly and justly. | _1_☐ | _2_☐ | _3_☐ | _4_☐ | _5_☐ |
| CRQ9 | I feel that my partner can be counted on to help me. | _1_☐ | _2_☐ | _3_☐ | _4_☐ | _5_☐ |
| CRQ10 | My partner and I try to discuss problems. | _1_☐ | _2_☐ | _3_☐ | _4_☐ | _5_☐ |
| CRQ11 | My partner and I express our feelings to each other. | _1_☐ | _2_☐ | _3_☐ | _4_☐ | _5_☐ |
| CRQ12 | We suggest possible solutions and compromises. | _1_☐ | _2_☐ | _3_☐ | _4_☐ | _5_☐ |
| CRQ13 | We threaten each other with negative consequences. | _1_☐ | _2_☐ | _3_☐ | _4_☐ | _5_☐ |
| CRQ14 | I call my partner names, swear at him or attack his character. | _1_☐ | _2_☐ | _3_☐ | _4_☐ | _5_☐ |
| CRQ15 | My partner calls me names, swears at me or attacks my character. | _1_☐ | _2_☐ | _3_☐ | _4_☐ | _5_☐ |

**Couples Relationship Satisfaction - CRS**

| **CRS:** Now we want to know your personal satisfaction in your relationship with your current partner. Based on your personal opinion, please share your feelings about it. | |
| --- | --- |
| CRS1 | How well does your partner meet your needs?  _1_☐ _2_☐ _3_☐ _4_☐ _5_☐  Poorly Average Extremely well |
| CRS2 | In general, how satisfied are you with your relationship?  _1_☐ _2_☐ _3_☐ _4_☐ _5_☐  Unsatisfied Average Extremely satisfied |
| CRS3 | How good is your relationship compared to most?  _1_☐ _2_☐ _3_☐ _4_☐ _5_☐  Poor Average Excellent |
| CRS4 | How often do you wish you hadn’t gotten into this relationship?  _5_☐ _4_☐ _3_☐ _2_☐ _1_☐  Never Average Very often |
| CRS5 | To what extent has your relationship met your original expectations?  _1_☐ _2_☐ _3_☐ _4_☐ _5_☐  Hardly at all Average Completely |
| CRS6 | How much do you love your partner?  _1_☐ _2_☐ _3_☐ _4_☐ _5_☐  Not much Average Very much |
| CRS7 | How many problems are there in your relationship?  _5_☐ _4_☐ _3_☐ _2_☐ _1_☐  Very few Average Very many |

**Religiosity - REL**

| **REL:** The following section contains 3 statements about religious belief or experience. Please mark the extent to which each statement is true or not true for you. | |
| --- | --- |
| REL1 | In my life, I experience the presence of the Divine (i.e., God).  _1_☐Definitely not true _2_☐ Tends not to be true _3_☐ Unsure _4_☐ Tends to be true _5_☐ Definitely true of me |
| REL2 | My religious beliefs are what really lie behind my whole approach to life.  _1_☐Definitely not true _2_☐ Tends not to be true _3_☐ Unsure _4_☐ Tends to be true _5_☐ Definitely true of me |
| REL3 | I try hard to carry my religion over into all other dealings in life.  _1_☐Definitely not true _2_☐ Tends not to be true _3_☐ Unsure _4_☐ Tends to be true _5_☐ Definitely true of me |

**Sexual Relationship Power - SRP**

| **SRP:** Please rate your level of agreement to the following statements. Mark the one box for each statement that best reflects your personal feelings. ***1=Strongly agree, 2=Agree, 3=Disagree, 4=Strongly disagree*** | | | | | |
| --- | --- | --- | --- | --- | --- |
| SRP1 | Under no circumstances would I ever leave my partner. | _1_☐ | _2_☐ | _3_☐ | _4_☐ |
| SRP2 | If my partner were to leave me, I would be in serious trouble. | _1_☐ | _2_☐ | _3_☐ | _4_☐ |
| SRP3 | If things were really bad with my partner, I would leave the relationship. | _1_☐ | _2_☐ | _3_☐ | _4_☐ |
| SRP4 | If my partner failed to meet my needs, I could easily find another partner. | _1_☐ | _2_☐ | _3_☐ | _4_☐ |
| SRP5 | My partner and I sit down and discuss important matters together. | _1_☐ | _2_☐ | _3_☐ | _4_☐ |
| SRP6 | My partner shows that he care about me. | _1_☐ | _2_☐ | _3_☐ | _4_☐ |
| SRP7 | If I suspect my partner is having an affair, I would talk with my partner. | _1_☐ | _2_☐ | _3_☐ | _4_☐ |
| SRP8 | I would consult with my advisors/friends if my partner was behaving badly. | _1_☐ | _2_☐ | _3_☐ | _4_☐ |
| SRP9 | When I need my partner's assistance, he is always there to help me. | _1_☐ | _2_☐ | _3_☐ | _4_☐ |
| SRP10 | I initiate sex with my partner when I want to have sex. | _1_☐ | _2_☐ | _3_☐ | _4_☐ |
| SRP11 | I am able to buy expensive items without my partner’s approval. | _1_☐ | _2_☐ | _3_☐ | _4_☐ |
| SRP12 | I have my own money to buy things I want. | _1_☐ | _2_☐ | _3_☐ | _4_☐ |
| SRP13 | My partner punishes me when he is angry with me. | _1_☐ | _2_☐ | _3_☐ | _4_☐ |
| SRP14 | When I disagree with my partner's relatives, my partner chooses their side over mine. | _1_☐ | _2_☐ | _3_☐ | _4_☐ |
| SRP15 | My partner is probably having sex with someone else. | _1_☐ | _2_☐ | _3_☐ | _4_☐ |
| SRP16 | If my partner was really angry with me, he might beat me. | _1_☐ | _2_☐ | _3_☐ | _4_☐ |

**HIV Risk and risk perception - RRP**

| **RRP:** The following questions are about your HIV sero-status and perception on the likelihood that you are at risk of getting HIV. Please answer accordingly. *YOUR RESPONSE IS CONFIDENTIAL!* | |
| --- | --- |
| RRP1 | On the scale 1 to 5, 1 being VERY LIKELY and 5 VERY UNLIKELY; please rate how likely you are at risk of getting HIV infection. _1_☐ _2_☐ _3_☐ _4_☐ _5_☐  _6_☐ *I’m HIV positive ….* ***Skip to RRP4*** |
| RRP2 | Do you think that your current sexual behaviour puts you at risk of HIV infection?  _1_☐ Yes _2_☐ No _3_☐ I don’t know |
| RRP3 | Do you think that your partner’s current sexual behaviour puts you at risk of HIV infection?  _1_☐ Yes _2_☐ No _3_☐ I don’t know |
| RRP4 | Have you ever had sexual intercourse with anyone other than your partner in the last twelve months? _1_☐ Yes _2_☐ No |
| RRP5 | How often do you use condom during your non-partner sexual intercourse?  _1_☐ Never _2_☐ Sometimes _3_☐ Usually but not always _4_☐ Always |
| RRP6 | How often do you use condom during sexual intercourse with your current partner?  _1_☐ Never _2_☐ Sometimes _3_☐ Usually but not always _4_☐ Always |

Finally, we would like to include your partner in the study as well. Can you invite him to participate? ☐ Yes ☐ No, why would you not invite him

**Thank you for your participation!**

Please return the questionnaire according to the instructions provided.
